# An apple a day: MdBPC2 transcription factor keeps the auxin away and causes dwarfing in *Malus domestica*

**DOI:** 10.1093/plcell/koad309

**Published:** 2023-12-12

**Authors:** Carlisle Bascom

**Affiliations:** Assistant Features Editor, The Plant Cell, American Society of Plant Biologists; Natural Resources and the Environment Department, University of New Hampshire, Durham, NH, 03824, USA

Managing plant height is imperative to the success of agriculture. Plant height is particularly important for tree crops such as pome fruits (e.g. apples, pears), where harvesting is challenging if the plants are too tall. Farmers manage fruit tree height in part by grafting shoots to dwarf rootstock. While horticultural research has focused on the productivity of trees grafted to dwarf rootstock (Kosina 2010), understanding the molecular underpinning of dwarfing in apples remains understudied. Myriad genes control plant size and development in *Arabidopsis thaliana*, including those encoding the BASIC PENTACYSTEINE (BPC) class of transcription factors (Monfared et al. 2011). However, whether BPCs regulate development in woody plants is an open question. In this issue, **Haiyan Zhao and colleagues** (Zhao et al. 2024) reveal some details of the genetic regulation of dwarfism in apple (*Malus domestica*) trees. Through genetics, bioinformatics, and biochemical assays, Zhao and colleagues elucidated a key molecular mechanism for plant dwarfism (see Figure).

**Figure.**
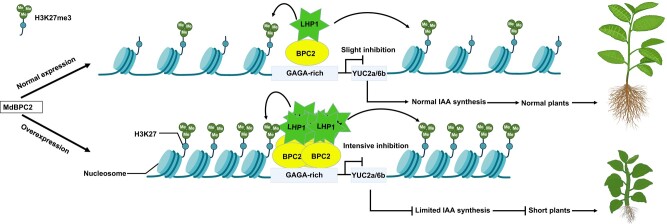
Moderate inhibition of auxin accumulation occurs when BPC2 recruits LHP1, which methylates histones in the *YUC2a/6b* loci. Dwarf rootstock results from increased levels of BPC2, which severely limits auxin biosynthesis. Adapted from Zhao et al. (2024), Figure 9.

Previous work has demonstrated the importance of BPC proteins in regulating plant height in *Arabidopsis* (Monfared et al. 2011). Therefore, the authors hypothesized that BPC proteins would also have an important role in *M. domestica* development. The authors identified 6 *MdBPC* gene*s* in the *M. domestica* genome and compared the expression of each in standard and dwarf rootstock. *MdBPC2* expression was elevated in dwarf rootstock and therefore became the focus of their study. To interrogate the functional connection between *MdBPC2* expression and plant height, the authors generated stable lines either overexpressing (OX) or silencing (RNAi) *MdBPC2*. Whereas the RNAi lines had no detectable phenotype, likely due to genetic redundancy, the OX plants were significantly shorter and thicker than the wild type—much like dwarf apple rootstock. By measuring the hormone content of the OX plants, the authors found that the shorter plants had half the auxin of wild type. Auxin, like other phytohormones, plays numerous roles in determining plant structure. These results gave the authors a clue to how BPC proteins regulate plant height.

As transcription factors, BPCs presumably function by affecting gene expression. Therefore, the authors performed RNA-seq with the OX plants and DNA-Affinity Purification (DAP)-seq with recombinant MdBPC2 protein to identify its targets. The authors cross-referenced the list of over 1,000 differently expressed genes with a list of 42 conserved auxin metabolism genes. This screening narrowed the candidate gene list to only a few, including 2 encoding flavin monooxygenases involved in auxin biosynthesis: *YUCCA(YUC)2a* and *6b*. The DAP-seq revealed that MdBPC2 binds to the *MdYUC2a* and *6b* promoters, and biochemical assays validated that MdBPC2 binds with the GAGA-Rich elements of *MdYUC2a* and *YUC6b* promoters and represses their expression (see Figure). These results comport with previous reports of BPCs being transcriptional repressors (Wu et al. 2020). The authors then sought to identify how MdBPC2 repressed *MdYUC2a* and *6b* expression.

Histone trimethylation (H3K27me3) results in repression of target genes (Cai et al. 2021). Here, the authors found that H3K27me3 enrichment was significantly increased at *MdYUC2a* and *6b* loci in OX plants. H3K27me3 modifications are facilitated by a large complex of proteins known as the polycomb group. The author used biochemical techniques to demonstrate that MdBPC2 interacts with the polycomb group member LIKE HETEROCHROMATIN PROTEIN1 (LHP1). Indeed, *MdYUC2a* and *6b* loci are enriched with MdLHP1 (see Figure). With this result, the authors proposed a straightforward model whereby MdBPC2 protein recruits LHP1 protein to the promoters of a subset of *YUCCA* genes to repress their expression, thereby reducing the amount of auxin produced. In dwarf rootstocks, increased *MdBPC2* expression results in much fewer MdYUC2a and 6b enzymes and shorter, auxin-deficient plants (see Figure). Moving forward, one can see *BPCs* as an attractive target for crop breeding programs where dwarf plants are the goal.

## Dive Curated Terms

The following phenotypic, genotypic, and functional terms are of significance to the work described in this paper:

BPC2 Gramene: AT1G14685
BPC2 Araport: AT1G14685

h3k27me3 CHEBI: CHEBI:85045
